# Matrix Metalloproteinases and Tissue Inhibitors of Metalloproteinases in Echinoderms: Structure and Possible Functions

**DOI:** 10.3390/cells10092331

**Published:** 2021-09-06

**Authors:** Igor Yu. Dolmatov, Vladimir A. Nizhnichenko, Lyudmila S. Dolmatova

**Affiliations:** 1 A.V. Zhirmunsky National Scientifc Center of Marine Biology, Far Eastern Branch, Russian Academy of Sciences, Palchevsky 17, 690041 Vladivostok, Russia; 0hm@mail.ru; 2 V.I. Il’ichev Pacific Oceanological Institute, Far Eastern Branch, Russian Academy of Sciences, Baltiyskaya 43, 690041 Vladivostok, Russia; dolmatova@poi.dvo.ru

**Keywords:** echinoderms, matrix metalloproteinase, tissue inhibitors of metalloproteinases, tensilin, development, regeneration, evolution

## Abstract

Echinoderms are one of the most ancient groups of invertebrates. The study of their genomes has made it possible to conclude that these animals have a wide variety of matrix metalloproteinases (MMPs) and tissue inhibitors of metalloproteinases (TIMPs). The phylogenetic analysis shows that the MMPs and TIMPs underwent repeated duplication and active divergence after the separation of Ambulacraria (Echinodermata+Hemichordata) from the Chordata. In this regard the homology of the proteinases and their inhibitors between these groups of animals cannot be established. However, the MMPs of echinoderms and vertebrates have a similar domain structure. Echinoderm proteinases can be structurally divided into three groups—archetypal MMPs, matrilysins, and furin-activatable MMPs. Gelatinases homologous to those of vertebrates were not found in genomes of studied species and are probably absent in echinoderms. The MMPs of echinoderms possess lytic activity toward collagen type I and gelatin and play an important role in the mechanisms of development, asexual reproduction and regeneration. Echinoderms have a large number of genes encoding TIMPs and TIMP-like proteins. TIMPs of these animals, with a few exceptions, have a structure typical for this class of proteins. They contain an NTR domain and 10–12 conservatively located cysteine residues. Repeated duplication and divergence of TIMP genes of echinoderms was probably associated with an increase in the functional importance of the proteins encoded by them in the physiology of the animals.

## 1. Introduction

### 1.1. Echinoderms

**Figure 1:**
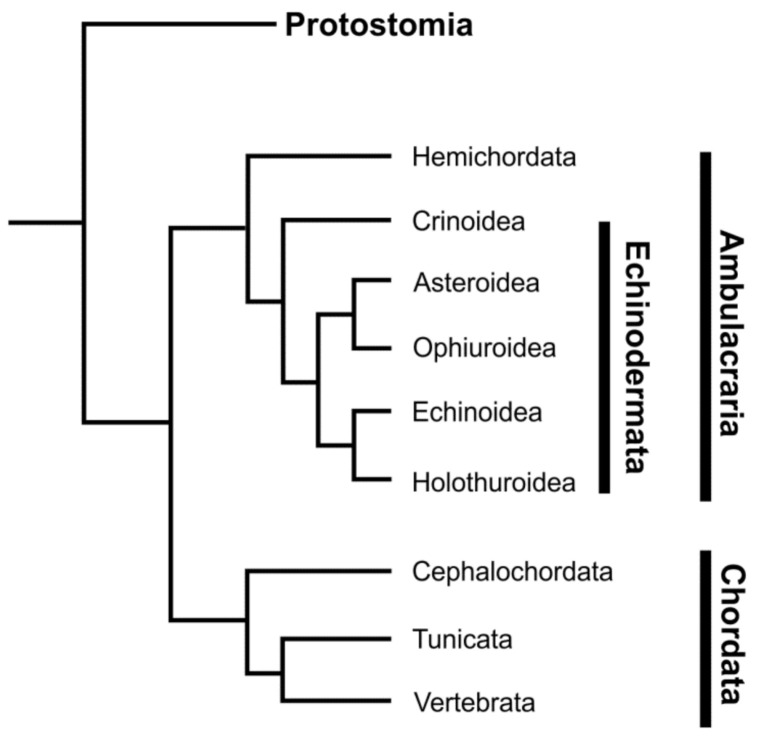
Phylogenetic tree showing the relationship of echinoderms to other animal groups.

Echinoderms are one of the most ancient groups of invertebrates. They most likely appeared in the Cambrian period, since several classes of these animals already existed in the early Ordovician, in particular rhombiferous and crinoids [1,2]. Phylogenetically, echinoderms are deuterostomes and, together with hemichordates, form the Ambulacraria group, which is the sister group to the chordates [3] (Figure 1).

Echinoderms are exclusively marine animals found in all regions of the world oceans in a wide range of depths, from shallow, intertidal zones to 5000 m or more. Most echinoderms are benthic organisms, though there are some swimming species [4,5], and some may be fully pelagic [6]. Modern species of echinoderms are grouped into five classes: Crinoidea (sea lilies and feather stars), Asteroidea (sea stars), Ophiuroidea (brittle stars or ophiuroids), Echinoidea (sea urchins), and Holothuroidea (holothurians or sea cucumbers). Despite the variety of structures, they all share radial symmetry and a pentameric body organization [7].

A significant part of the echinoderm’s body wall is made up of a calcified connective tissue forming a unique structure, the stereom. As a result, the majority of echinoderms, with the exception of the holothurians, have a solid cover which protects their internal organs from external threats. Nevertheless, these animals can autotomize various parts of the body and reproduce asexually by transverse fission [8,9,10,11,12]. The ability to separate body parts is carried out by changing of the mechanical properties of the connective tissue referred to as mutable collagenous tissue (MCT) [13] or catch connective tissue [14]. An extracellular matrix (ECM) with MCT properties can form various anatomical structures, in particular, ligaments and connective body wall tissue [8,15].

Another widely known peculiarity of echinoderms is their regenerative abilities [16,17,18,19]. They can restore small body appendages, such as tentacles, tube feet, cirri and spines, and also heal cutaneous wounds. Echinoderms can regenerate all their internal organs [16,17,20]. In addition, these animals can not only regenerate large body parts, such as arms, but they can also entirely regenerate themselves from a small fragment after being cut into two or three parts [20,21,22,23,24,25,26,27,28].

In echinoderms, the connective tissue and its transformation play an important role not only in autotomy and fission, but also during regeneration. In the latter case, the significance of ECM remodeling is determined by the fact that many organs of these animals are epithelial formations containing a large amount of connective tissue. This is especially characteristic of the holothurians. The connective-tissue base (connective-tissue thickening) is formed first in the regeneration of the internal organs, and then certain cells or epithelia migrate into it [19,29,30,31,32,33].

### 1.2. Components of Connective Tissue of Echinoderms

Echinoderms have genes encoding a variety of ECM components—collagens, proteoglycans, and glycoproteins [34,35,36,37,38,39]. These proteins are common in most multicellular animals [40]. At the same time, there are differences in the connective tissue between echinoderms and vertebrates. In particular, one of the main components of vertebrate ECM is elastin, the fibers of which are formed due to the polymerization of tropoelastin [41]. No homologs of *tropoelastin* were found in the genomes of crinoid *Anneissia japonica* (Müller, 1841) and sea urchin *Strongylocentrotus purpuratus* (Stimpson, 1857). There are no its transcripts in the holothurian transcriptomes [35,42,43].

Another difference in ECM echinoderms is the lack of tenascins and fibronectins [44]. These proteins play an important role in the structural integrity of connective tissues in vertebrates [45,46]. Some holothurians have been found to blast transcripts as tenascin-like proteins [35,47]. These contigs encode the domains characteristic of tenascins—FBG and TILa. Nevertheless, according to the opinion of Hynes [48], all these domains are ancient in origin and are found in many animals; however, in a combination characteristic of tenascins, they appear only in chordates. Thus, the absence of such proteins as tropoelastin, fibronectins, and tenascins indicates significant differences in the organization of connective tissue in echinoderms and chordates.

The basis of the extracellular matrix of any animal is collagens. Echinoderms have representatives in almost all major groups of these proteins—fibril-forming collagens, fibril-associated collagens with interrupted triple helices (FACIT), network-forming collagens, and multiplexins [34,35,49]. Moreover, proteoglycans and glycoproteins are important components of connective tissue. They perform many functions, mediating adhesion, proliferation, differentiation and migration of different types of cells [50,51,52]. Echinoderms have orthologs of genes encoding various proteoglycans: *aggrecan*, *syndecan*, *glypican*, *bamacan* (*structural maintenance of chromosomes 3*), *perlecan* (*basement membrane-specific heparan sulfate proteoglycan core protein-like*), *betaglycan* and *Secreted modular calcium-binding protein 1* [34,35]. In addition, they have genes for various glycoproteins—*laminins*, *nidogens*, *fibrillins*, *fibulins*, *agrin*, *dystroglycan*, and *thrombospondins* [34,35,53]. Proteins encoded by these genes, together with collagens and perlecan, are included in the basic set of “the basement membrane ECM toolkit”, common to all Bilateria [34]. The connective tissue also contains oligo- and polysaccharides such as N-glycans, glycogen, hyaluronic acid and heparan sulfate [54,55,56,57,58].

### 1.3. Proteins Modifying Connective Tissue

As already indicated, ECM remodeling plays a large role in various physiological processes in echinoderms. In this regard, these animals must have a set of various enzymes that modify connective tissue. First of all, it should be noted the proteins that synthesize the main component of the ECM, collagen. The formation of collagen fibrils occurs due to the activity of a special enzyme—lysyl oxidase (LOX) [49]. Echinoderms also have *lox* orthologs [35]. In addition, they have genes that code for enzymes that synthesize and degrade polysaccharides—*hyaluronidase*, *chondroitin sulfate synthase* and *heparanase* [35].

A wide variety of proteinases capable of degrading ECM proteins have now been found in echinoderms. They are serine, cysteine, aspartyl, and metal peptidases and their inhibitors [35,59,60,61,62,63,64,65,66,67]. For example, the genome of the sea urchin *S. purpuratus* contains approximately 240 metalloprotease genes [62]. They represent all 23 families expressed in vertebrates. Among them are the genes encoding BMP-1/tolloid (astacins), matrix metalloproteinases (MMPs), a disintegrin and metalloproteinases (ADAMs), and tissue inhibitors of metalloproteinase (TIMPs). The transcriptome of the holothurian *Cladolabes schmeltzii* (Ludwig, 1875) contains the products of at least nine genes of serine proteases *proprotein convertase subtilisin/kexin type 9* (*PCSK9*) belonging to the subtilases family [35]. In addition, this animal has *furin* and *plasminogen*.

Of the cysteine proteases in echinoderms, the genes *cathepsin B* and *cathepsin L* are found [35,61]. Cathepsins B and L are lysosomal proteinases and are located in cells. However, they can be secreted in the ECM and digest connective tissue proteins [68,69]. In holothurian *Apostichopus japonicus* (Selenka, 1867) cathepsin L-like protein is found in the epidermis and the cells located in the outer layer of the dermis [61]. It is assumed that it can participate in the processes of autolysis of the connective tissue of the body wall.

One of the main ways to change the properties of ECM is the degradation of its proteins by MMPs [70]. Moreover, the MMPs are involved in many other biologic processes, such as, development, regeneration, cell proliferation, apoptosis, cell differentiation and migration [70,71,72,73,74]. The activity of MMPs is modulated by their natural inhibitors, TIMPs [74,75,76,77]. Echinoderms also have MMPs and TIMPs [19,35,62,78]. At least 26 genes encoding MMPs were found in the genome at the sea urchin *S. purpuratus* [62]. Unlike mammals with only four *TIMP*s, some species of echinoderms can have up to 45 *TIMP* genes [78]. In the holothurians, it was shown that various *MMPs* and *TIMPs* were expressed in asexual reproduction and regeneration [19,35,64,79]. MMP blocking leads to a slowdown or full stop of morphogenesis [63,65,80,81].

Based on the interaction of MMPs and TIMPs, a mechanism has been proposed to explain the changes in the properties of the MCT [82]. Achieving greater stiffness or, on the contrary, more plasticity in the connective tissue occurred as a result of the interaction of three protein groups: MMPs, TIMPs, and cross-link complexes connecting collagen fibrils to one another. As the activity of TIMPs increase, MMPs are blocked. As a result, cross-links are formed between collagen fibrils while the MCT strengthens. Conversely, an increase in the MMPs concentration or activity in connective tissue leads to the destruction of the cross-link complexes. This destruction enables collagen fibrils to slide along one another, which brings the MCT into a compliant state.

However, a detailed analysis of MMPs and TIMPs has not been carried out in echinoderms. In this regard, this paper is devoted to the characteristics of these proteins from different classes of the phylum Echinodermata. For the analysis, we used the genomes of representatives of four echinoderm classes: crinoid *Anneissia japonica* (PRJNA615663), sea star *Patiria miniata* (Brandt, 1835) (PRJNA683060), sea urchin *S. purpuratus* (PRJNA13728), holothurian *Apostichopus japonicus* (PRJNA354676). Hereinafter accession numbers of genomes and nucleotide sequences from the NCBI database (https://www.ncbi.nlm.nih.gov, accessed on 15 March 2021) are shown in parentheses. The sequences used for analysis are presented in Supplementary Files 1 and 2. A list of protein names and accession numbers of nucleotide sequences of the corresponding transcripts from the NCBI database (https://www.ncbi.nlm.nih.gov, accessed on 15 March 2021) are presented in Supplementary File 3.

## 2. Matrix Metalloproteinases of Echinoderms

### 2.1. Domain Structure

It was shown that the number of MMPs varies in different species of echinoderms. The genome of crinoid *Anneissia japonica* revealed 22 *MMP* genes, in the sea star *P. miniata* 20 *MMPs*, the sea urchin *S. purpuratus* has 22 *MMPs*, and the holothurian *Apostichopus japonicus* has 17. This is comparable to the number of *MMPs* in vertebrates, which have 25–33 *MMP* genes.

A study of the domain structure of echinoderm MMPs shows that they have a composition typical of this type of proteases. A signal peptide is located at the N-terminus, followed by a propeptide domain. This often includes the proteoglycan-binding domain (PGBD). The propeptide domain is followed by the catalytic domain. Many echinoderm MMPs have several hemopexin-like repeats at the C-terminus of the molecule.

**Figure 2:**
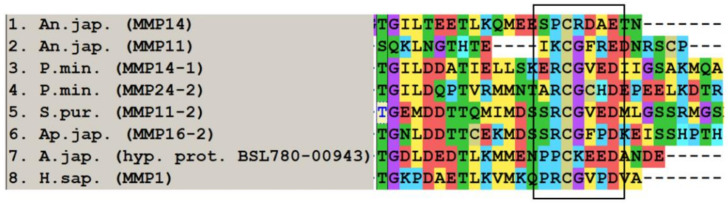
MUSCLE (MegaX) alignment of cysteine switch sequences (boxed area) of some MMPs of echinoderms.

**Figure 3:**
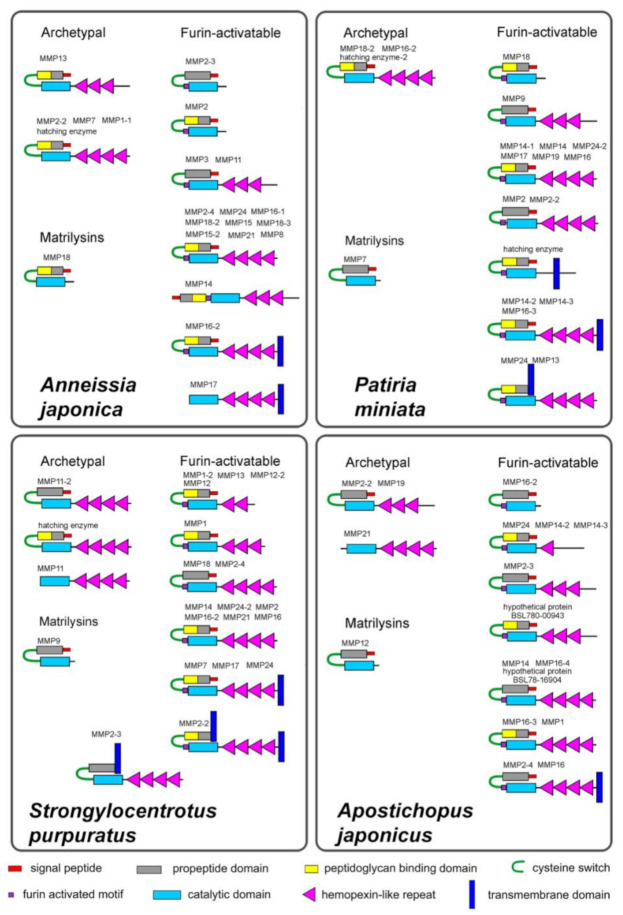
Scheme of structure of the matrix metalloproteinases of echinoderms. The domain structure was revealed using the Pfam (http://pfam.xfam.org/, accessed on 15 March 2021), Blast NCBI, and Smart (http://smart.embl-heidelberg.de/#, accessed on 15 March 2021) programs. In addition, SignalP-5.0 Server (http://www.cbs.dtu.dk/services/SignalP, accessed on 15 March 2021) and Phobius (https://phobius.sbc.su.se/index.html, accessed on 15 March 2021) were used to more accurately determine the presence of a signal peptide and transmembrane domains in a protein molecule.

The propeptide domain of MMPs ends with a conserved sequence, the cysteine switch. The cysteine contained in the cysteine switch interacts with the zinc of catalytic domain and inactivates proteolytic activity of MMP [83]. The sequence of a cysteine-switch motif in echinoderm MMPs is very often quite different from that of vertebrates (PRCGXPD, [84] Figure 2). The CG sequence may be the most conservative. Quite often first P (proline) is replaced by threonine (T), serine (S), alanine (A), glutamic acid (E), glutamine (Q), or lysine (K). In some cases, the propeptide ends with a sequence that is completely different from a cysteine-switch motif. For example, the crinoid *Anneissia japonica* has MMPs with SPCRDAE (MMP14, XM_033271712.1) and IKCGFRE (MMP11, XM_033270068.1) sequences (Figure 2). In some echinoderm MMPs, the cysteine switch is absent. Immediately upstream of the catalytic domain in most echinoderm MMPs is the furin activated motif RX[K/R]R. Additionally, a transmembrane domain can be located at the N- or C-terminus of the molecule. The schemes of the MMP structure in representatives of different echinoderm classes are presented in Figure 3.

Judging by the domain structure, most proteinases are synthesized in an inactive form, zymogen, which is then activated by removing the propeptide domain. The catalytic domain of echinoderm MMPs contains the characteristic zinc binding motif HEXXHXXGXXH [85]. This shows that their mechanism of protein cleavage is similar to that of vertebrate MMPs.

The presence of a PGBD-like domain at the N-terminus of a number of echinoderm MMPs indicates that these MMPs, like similar vertebrate MMPs, can bind to proteoglycans and catalyze extracellular matrix degradation. However, not all echinoderm MMPs have PGBD-like domains (Figure 3). It is possible that the function of MMPs without these domains is not related to ECM transformation. They may participate in the regulation of cell migration or differentiation through the destruction of certain proteins such as integrins or receptors.

Thus, echinoderm MMPs have a standard domain structure similar to that of vertebrate MMPs [71,86]. Four different groups are distinguished by their structure in mammals: archetypal MMPs, matrilysins, gelatinases, and furin-activatable MMPs. MMPs in echinoderms can be divided into three groups: archetypal MMPs, matrilysins, and furin-activatable MMPs (Figure 3).

A number of echinoderm MMPs are known to be capable of degrading denatured collagen (gelatin) [63,65,81,87,88,89]. However, typical gelatinases similar in structure to vertebrate gelatinases (MMP2 and MMP9) do not seem to exist in echinoderms. In MMP2 and MMP9, the catalytic domain contains fibronectin-like repeats [86] which gives the ability to bind and degrade gelatin, suggesting that these enzymes play an important role in collagen remodeling of the extracellular matrix [90]. The NCBI database shows a transcript of the holothurian *Apostichopus japonicus* (MH348178.1), designated here as *MMP2-1*. Analysis of its putative amino acid sequence shows that this proteinase also contains a fibronectin-like repeats insertion in the catalytic domain. However, phylogenetic analysis showed that this protein is clustered together with fish MMP2 and separate from all MMPs of echinoderms (see below). In this regard, this contig is most likely the result of contamination of a holothurian tissue samples with vertebrate mRNA, most likely from bony fish *Sinocyclocheilus rhinocerous* Li & Tao, 1994.

#### 2.1.1. Archetypal MMPs

Echinoderm MMPs, which can be assigned to the group of archetypal MMPs, have a similar structure in all classes (Figure 3). The molecules of these proteinases have a signal peptide, a propeptide domain with a cysteine switch, a catalytic domain, and 3–4 hemopexin-like repeats. In the holothurian *A. japonicus*, PGBD is absent in the propeptide domains of all three archetypal MMPs. One PGBD is missing from two MMPs in the sea urchin *S. purpuratus*. In *Anneissia japonica* and *P. miniata*, all archetypal MMPs contain PGBD.

MMPs with a missing propeptide domain were previously identified in the genome of *S. purpuratus* [62]. We also detected MMPs of this structure in this species. In addition, the holothurian *Apostichopus japonicus* also have one MMP that lacks a propeptide domain. These MMPs are classified as archetypal MMPs because they lack the furin activated motif and have hemopexin-like repeats.

#### 2.1.2. Matrilysins

The examined species of echinoderms each have one MMP, which can be referred to as the matrilysins group. They have a similar structure and consist of a signal peptide, a propeptide domain with a cysteine switch, and a catalytic domain.

#### 2.1.3. Furin-Activatable MMPs

The vast majority of the studied echinoderm MMPs contains furin activated motifs and, accordingly, they can be referred to as the group of furin-activatable MMPs. Like in vertebrates [86], this group combines proteases with different domain structures. Most of them are structurally similar to archetypal MMPs and matrilysins. They differ in the number of hemopexin-like repeats and the presence or absence of PGBD (Figure 3).

In addition, transmembrane domains are detected in some echinoderm MMPs. Because the N- and C-terminus of the MMP molecule has a large number of hydrophobic amino acids (valine, isoleucine, leucine, alanine, phenylalanine), determining the presence of transmembrane domains in echinoderms should be approached with caution. In this article, we have cited the transmembrane domain only when its presence was confirmed by several programs. In the echinoderm species studied, transmembrane domains are found at both the N- and C-terminus of the MMP molecule.

**Figure 4:**
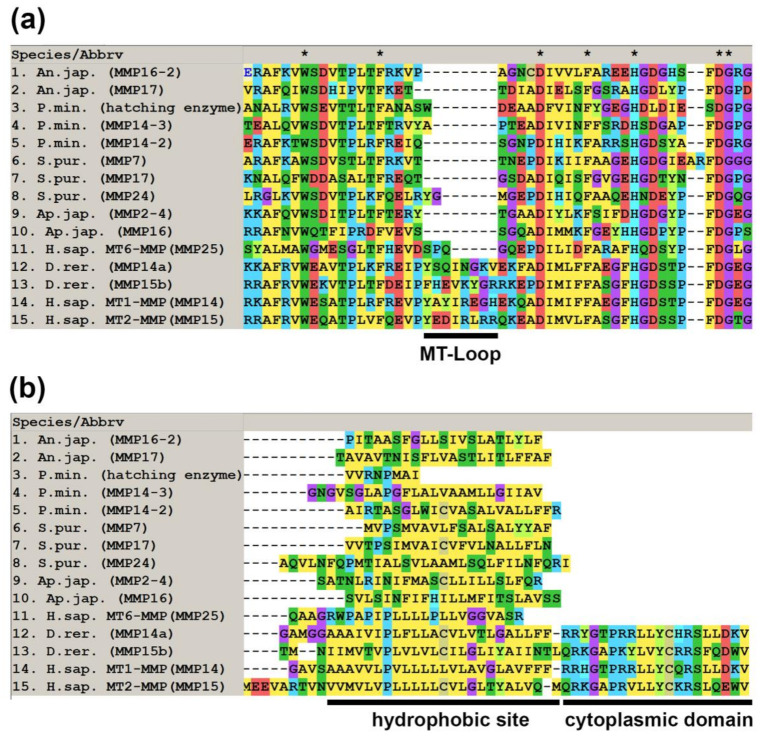
MUSCLE (MegaX) alignment of amino acid sequences of MMPs with a posterior transmembrane domain of echinoderms and vertebrates. (**a**) Catalytic domain with MT-Loop; (**b**) C-terminus of MMPs.

In each echinoderm species studied, 2-3 MMPs were identified with a posterior transmembrane domain (Figure 3). These MMPs differ in their structure from the type I transmembrane MT-MMPs (MT1-, MT2-, MT3- and MT5-MMPs) of vertebrates. In the latter, the catalytic domain contains a characteristic sequence of 8-9 amino acids, an MT-Loop [91,92,93]. It is assumed that MT-Loop plays a major role in the regulation of type I transmembrane MT-MMPs function [92]. In echinoderms, no such sequence is present in any of the studied MMPs (Figure 4). In addition, all vertebrate’s type I transmembrane MT-MMPs have a DUF3377 domain at the C-terminus. It is absent in echinoderm MMPs. At the same time, the hydrophobic site at the C-terminus in echinoderm MMPs is similar to that of vertebrate glycosylphosphatidylinositol (GPI)-anchored type MT-MMPs (MT4- and MT6-MMPs) [94,95,96] (Figure 4). Thus, echinoderm MMPs with a transmembrane domain at the C-terminus are more similar to (GPI)-anchored MT-MMPs. However, this assumption requires further verification.

Another group of echinoderm MMPs is proteinases in which the transmembrane domain is detected at the N-terminus of the molecule. In this respect, they are similar to type II transmembrane MMPs (MMP23) of vertebrates [97,98]. Such MMPs were found in the sea star *P. miniata* and the sea urchin *S. purpuratus* (Figure 3). In contrast to the vertebrate MMP23s, they all contain cysteine switch and hemopexin-like repeats. Moreover, the *S. purpuratus* proteinase does not have a furin activated motif. Thus, echinoderm MMPs with a transmembrane domain at the N-terminus are significantly different not only from the type II transmembrane MMPs of mammals but also from those of lower vertebrates which lack a C-terminal cysteine-rich toxin-like and an immunoglobulin-like cell adhesion molecule domains [97,99] and are obviously not their homologs. Nevertheless, the possible presence of a transmembrane domain at the N-terminus of the molecule suggests that these echinoderm MMPs in question may function intracellularly, as do MMP23 in mammals [98].

In addition to MMPs with a single transmembrane domain, it has been noted that the sea urchin *S. purpuratus* MMP2-2 (XM_776482.5) has transmembrane domains at both ends of the molecule (Figure 3). It is not clear whether this is an error in the detection of these domains by the softwares used, or if this is indeed the case. Since this MMP has a cysteine switch and a furin activated motif, it is obvious that its activation is due to the removal of the propeptide along with a possible anterior transmembrane domain. This structure probably reflects the peculiarities of the biosynthesis and/or secretion of this proteinase. In any case, this fact deserves further investigation because in mammals there are functioning proteins with transmembrane domains at both ends of the molecule [100].

### 2.2. Evolution of MMPs of Echinoderms

**Figure 5:**
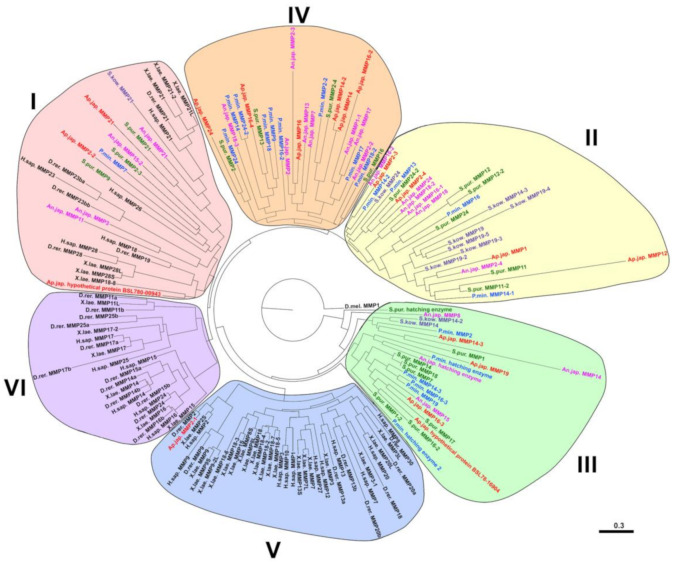
Phylogenetic tree showing the relationships of MMPs of vertebrates, hemichordates, and echinoderms. Crinoids (*Anneissia japonica*)—pink color, asteroids (*Patiria miniata*)—blue color, echinoids (*Strongylocentrotus purpuratus*)—green color, holothurians (*Apostichopus japonicus*)—red color, hemichordates (*Saccoglossus kowalevskii*)—deep-blue color, vertebrates—black color. Determination of conserved regions of the putative amino acid sequences was carried out using the Gblock program. Construction of the phylogenetic tree was done using the MrBayes/Maximum Likelihood algorithm and the online service CIPRES (http://www.phylo.org, accessed on 15 March 2021). The nucleotide and amino acid sequences were analyzed using the Partitionfinder 2.1.1 [101,102]. The trees were visualized in the FigTree program.

In spite of the clear similarity of MMP domain organization between vertebrates and echinoderms, on the phylogenetic tree most echinoderm MMPs is clustered separately from vertebrate MMPs (Figure 5). This is apparently because the *MMP* genes of the last common ancestor of Chordata and Ambulacraria underwent substantial duplication and divergence following separation of the two groups [62]. Phylogenetic analysis shows that three groups can be distinguished among echinoderm MMPs, which differ in their proximity to vertebrate MMPs and, consequently, to ancestral forms of MMPs (Figure 5). The first group contains the MMPs of all the studied species of the deuterostomes. It was probably formed as a result of duplication and divergence of several ancestral genes. This includes such mammalian MMPs as MMP18, MMP21, MMP23, MMP26, MMP28. Apparently, the *MMP* genes of echinoderms from this group can be considered their orthologs.

The second group unites only the MMPs of representatives of Ambulacraria (Figure 5). Apparently, it formed after the separation of the chordates. The third group of MMPs contains mainly proteases of echinoderms and it is located closest to the base of the phylogenetic tree. This probably indicates that this group combines the most conserved MMPs, which diverged the least during the evolution of Ambulacraria. The fourth group contains only echinoderm MMPs. These proteases apparently duplicated and diverged after the separation of Echinodermata and Hemichordata. Thus, the MMPs belonging to the II-IV groups have no direct homology with any of the mammalian MMPs.

In the given phylogenetic tree, the fifth and sixth groups unite vertebrate MMPs. Interestingly, the MMP2-1 of holothurian *Apostichopus japonicus* (MH348178.1) clusters with vertebrate MMP2 and is located on the same branch as MMP2 of *Danio rerio* (Figure 5). As indicated above, this sequence most likely does not belong to holothurian *A. japonicus*.

**Figure 6:**
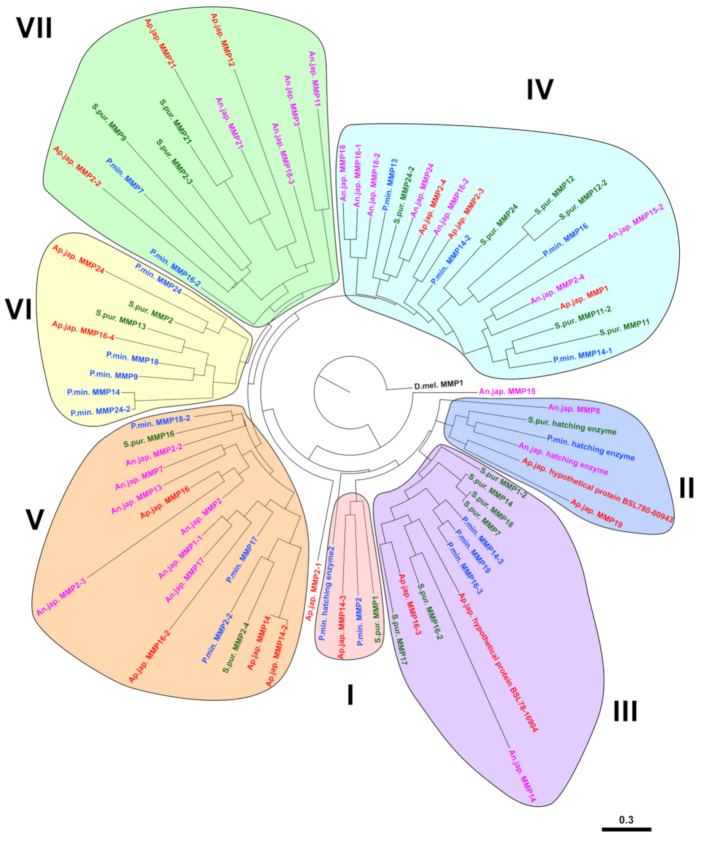
Phylogenetic tree showing the relationships of MMPs of echinoderms. Crinoids (*Anneissia japonica*)—pink color, asteroids (*Patiria miniata*)—blue color, echinoids (*Strongylocentrotus purpuratus*)—green color, holothurians (*Apostichopus japonicus*)—red color. Determination of conserved regions of the putative amino acid sequences was carried out using the Gblock program. Construction of the phylogenetic tree was done using the MrBayes/Maximum Likelihood algorithm and the online service CIPRES (http://www.phylo.org, accessed on 15 March 2021). The nucleotide and amino acid sequences were analyzed using the Partitionfinder 2.1.1 [101,102]. The trees were visualized in the FigTree program.

At present, the identification of echinoderm MMPs is performed solely on the basis of the proximity of their amino acid or nucleotide sequence to other proteases in the NCBI database. However, as indicated above, the bulk of these animals’ MMPs are not orthologous to vertebrate MMPs. Therefore, it does not make sense to draw conclusions about the homology and, on this basis, about the similarity of the functions of these proteinases. The constructed phylogenetic tree of MMPs of echinoderms identifies 7 large groups of proteinases, each of which, if necessary, can be divided into subgroups (Figure 6). This tree shows that within the phylum Echinodermata, the MMPs are also highly divergent. It is well observed that there is no correspondence between the names of the MMPs (the most likely homologues in BLAST) and the evolutionary relationship of the respective proteases. For example, MMPs of the holothurian *A. japonicus* MMP2-2 (MRZV01000410.1) and MMP2-3 (MRZV01000538.1), which by NCBI are “homologs”, are located in different parts of the tree. Conversely, sequences located on the same branch of the phylogenetic tree and which are very likely orthologous, have different designations, as, e.g., MMPs of *Anneissia japonica* MMP18 (XM_033252622.1) and MMP16-1 (XM_033252624.1) from group IV. Thus, the classification of MMPs of echinoderms requires revision and additional research.

### 2.3. Substrate Specificity and Function

Sea urchins are the most investigated model objects among echinoderms. Sea urchin MMPs with collagen-gelatinase activity were first found in developing embryos [103,104,105,106]. They are hypothesized to regulate the processes of hatching, gastrulation and hyaline layer development, as well as the growth of spicules. The identified gelatinases hydrolyze their own collagen as well as type I collagen from rat tails, but are not active against casein.

The most studied are the hatching enzymes (HEs). These proteases are found not only in sea urchins, but also in other echinoderms. On the phylogenetic tree, they cluster together and probably represent a separate group of echinoderm MMPs (Figure 5 and Figure 6). These proteases are expressed at the late blastula stage and are necessary for the dissolution of the fertilization envelope and release the embryo [106]. The described properties of HE6 were from *Paracentrotus lividus* (Lamarck, 1816) and the cloned *HE6* transcript (X65722.1) [107,108,109,110]. This proteinase had a typical MMP structure. The signal peptide was located at the N-terminus, followed by the propeptide domain, catalytic domain, and hemopexin-like repeats. HE6 by its domain structure can be attributed to an archetypal MMP, because it does not contain a furin-activated motif. This proteinase was able to hydrolyze dimethyl casein [107]. Its activity was completely blocked by 20 mM EDTA. This inhibition was irreversible and activity was not restored by the addition of Ca^2+^ ions.

*HE6* transcripts were not detected in unfertilized eggs [108]. This indicates that mRNAs of *HE6* do not belong to the maternal set of mRNAs. For the first time, *HE6* transcripts have been detected in blastula cells at the 128-cell stage [109]. The number of *HE6* transcripts then increases until the pre-hatching blastula stage (250 cells) and then returns to a very low level after hatching. *HE6* expression occurs only in the blastomeres of the animal part of the embryo, corresponding to the presumptive ectoderm. From the mesenchyme blastula stage, *HE6* mRNAs are not detected, which is probably due to rapid mRNA degradation.

HE, envelysin, (AB000719.1) demonstrated a similar structure and properties in the sea urchin *Hemicentrotus pulcherrimus* (A. Agassiz, 1864) [111]. This MMP was completely inhibited by alpha 2-macroglobulin and the chelating agents EDTA, EGTA, and 1,10-phenanthroline and was slightly inhibited by chymostatin and pepstatin [104]. A HE with similar properties and functions has also been found in sea stars [112,113]. In addition, various proteinases capable of degrading collagen and gelatin have been found in the eggs and developing embryos of the sea urchin *S. purpuratus* [87,88,89,114,115,116,117,118,119,120,121,122]. All of them show properties similar to those of the MMPs. However, the genes encoding these proteinases, as well as their domain structure, have not been established.

MMP probably plays a role in the early development of sea urchins [123]. The *SpMMP14* gene (XM_030985841.1) is expressed in unfertilized oocytes and then in embryonic cells at the mesenchymal blastula stage in *S. purpuratus*. At the beginning of gastrulation, the transcripts of this gene are found in the blastomeres on the animal and vegetal poles of the larva, and later are retained only at the animal pole. Expression of *SpMMP16* (Sp-Mt1-4/MmpL7, NM_001033648.1) begins at the blastula stage of *S. purpuratus*. The transcripts are localized in the area of the vegetal pole. Subsequently, its expression is limited to cells of the secondary mesenchyme.

MMPs participate in the formation of the larval skeleton in sea urchins. It was shown that during the cultivation of primary mesenchymal cells and embryos of *S. purpuratus* in the presence of metalloprotease inhibitors, inhibition of spiculogenesis occurs [124]. When the inhibitor was added at a stage where a small triradial skeleton had already formed, the growth of spicules was blocked. Proteomic analysis shows that the matrix of skeletal elements of *S. purpuratus* contains several MMPs [125]. The most numerous were Sp-Mmp18/19L3 (XM_030980004.1), Sp-Mmp18/19L6 (XM_030980002.1), Sp-Mmp18/19L5 and Sp-Mmp18/19L4 (XM_783693.4), Sp-Mt1-4/MmpL5 (XM_786507.5), Sp-Mt1-4/MmpL6 (XM_786523.5), Sp-Mt1-4/MmpL7 (NM_001033648.1), and Sp-Mt5/MmpL2 (XM_003725508.3). Our analysis shows that most of these MMPs are furin-activatable MMPs and have a standard domain structure represented by a PGBD propeptide, a catalytic domain, and hemopexin-like repeats. Two proteinases (Sp-Mmp18/19L3 and Sp-Mt5/MmpL2) contain transmembrane domains at the C-terminus.

Studies of the sea urchin *P. lividus* revealed two MMP genes expressed in the skeletogenic cells [126]. One of them, *Pl-MmpL5* (ortholog of *Sp-Mt1-4/MmpL5* of *S. purpuratus*) expressed at the blastula stage (10 h post fertilization, hpf). Transcripts of another gene, *Pl-MmpL7* (ortholog *Sp-Mt1-4/MmpL7* of *S. purpuratus*) are first found at the beginning of gastrulation (~20 hpf), and after 24 hpf they are detected at the skeletogenic lateral cell clusters. The expression level of both genes depends on VEGF-signaling as it decreases significantly when the VEGF receptor (VEGFR) is inhibited [126]. Thereafter, *Pl-MmpL5* and *Pl-MmpL7* are expressed at the tips of the growing rods. Moreover, VEGF signaling controls their activity only at the post-oral and anterolateral rods. The expression of *Pl-MmpL5* and *Pl-MmpL7* at the tips of the body rods does not change upon inhibition of VEGFR, which indicates the regulation of these genes by another regulatory system [126].

In adult echinoderms, MMPs also play an important role in various processes. In particular, during asexual reproduction by transverse fission, local softening and lysis of the connective tissue are necessary. The holothurian *Apostichopus japonicus* has MMPs that are capable of completely degrading body wall collagen [127]. Analysis of the transcriptome of the holothurian *C. schmeltzii* in the process of division revealed the presence of transcripts of eight MMP genes [35]. However, in this species, no differences were found in the qualitative composition of the *MMPs* between normal and dividing individuals. Probably, if MMPs are involved in the mechanisms of asexual reproduction, then during fission it is not the spectrum of proteinases that changes, but the level of their activity.

During regeneration, a large number of *MMP* genes are expressed in echinoderms [19,35,36,38,39,128]. Their activation is possibly induced by high levels of reactive oxygen species (ROS) [71,129], which are produced by echinoderm cells in response to damage [19]. In *A. japonicus*, two *MMP* genes have been described that are expressed during the regeneration of internal organs—*ajMMP-2* (*MMP2-4*, KX372219.1) and *ajMMP-16* (*MMP16-3*, KX372220.1) [64]. No transcripts of these genes were found in the intact intestine. Only 1-2 h after evisceration did *ajMMP-2* and *ajMMP-16* begin to be expressed. The maximum number of their transcripts occurred at 6 h and 24 h after injury, respectively. Proteinases were observed in the esophagus remnant 3 and 7 days after evisceration. Moreover, the distribution of these MMPs in the digestive system was different. In the luminal epithelium, only ajMMP-2 was localized, while ajMMP-16 was found in all tissues of the esophagus. Differences in the expression of the genes *ajMMP-2* and *ajMMP-16* and the distribution of proteins encoded by them indicate different functions of these proteinases in intestinal regeneration in holothurians. Since transcrips of *ajMMP-2* and *ajMMP-16* are detected only at the initial stage of regeneration, ajMMP-2 and ajMMP-16 are probably involved in the degradation of the esophageal ECM and dedifferentiation of coelomic epithelial cells and enterocytes [19]. In addition, it is possible that these proteinases regulate the interaction between ECM components and growth factors due to proteinolysis of ECM proteins and other biological molecules [64].

In the holothurian *Eupentacta fraudatrix* (D’yakonov & Baranova in D’yakonov, Baranova & Savel’eva, 1958)*,* several proteinases with gelatinase activity were found during the regeneration of various internal organs. Four proteinases with molecular masses of 132, 58, 53, and 47 kDa were detected in the digestive system [63]. Zymographic assay revealed different lytic activities of the proteinases during intestine regeneration. The 132 kDa proteinase showed the highest activity at the first stage. During morphogenesis (stages 2–4 of regeneration), the highest activity was measured for the 53 and 58 kDa proteinases. A similar set of proteinases was found during the regeneration of the body wall [65,80]. Inhibition of MMPs with GM6001 completely stopped the restoration of damaged organs.

Among these gelatinases, two have been identified so far—53, and 47 kDa proteinases (Shabelnikov, personal comm.). The first one, 53 kDa (GHCL01011560.1), is an archetypal MMP in its domain structure. The propeptide domain of this MMP does not contain PGBD. Four hemopexin-like repeats are located after the catalytic domain. The second, 47 kDa (GHCL01010993.1), is Ef-MMP16 [79]. This is a furin-activatable MMP. Its propeptide domain does not contain PGBD, and four hemopexin-like repeats are located at the C-terminus. It was shown that *Ef-MMP16* expresses during gut regeneration in holothurian *E. fraudatrix* [79]. Its transcripts were found in the coelomic epithelium of the mesentery and gut anlage. It is possible that this proteinase is involved in the regulation of migration and/or proliferation of coelomic epithelial cells.

## 3. Tissue Inhibitors of Metalloproteinases

### 3.1. Domain Structure

TIMPs are natural inhibitors of MMPs. Unlike vertebrates, echinoderms have a large number of genes encoding TIMPs and TIMP-like proteins [35,78]. In some species, the number of such genes can reach 45 [78]. Most of the studied TIMPs of echinoderms have a standard structure similar to that of TIMPs of other animals [35,78]. Only one domain is identified in them—the NTR domain, which is characteristic of this class of proteins. Most of the studied TIMPs of echinoderms contain 10–12 conserved cysteine residues, which probably form the tertiary structure of the molecule (Supplementary File 2) [35,78].

**Figure 7:**
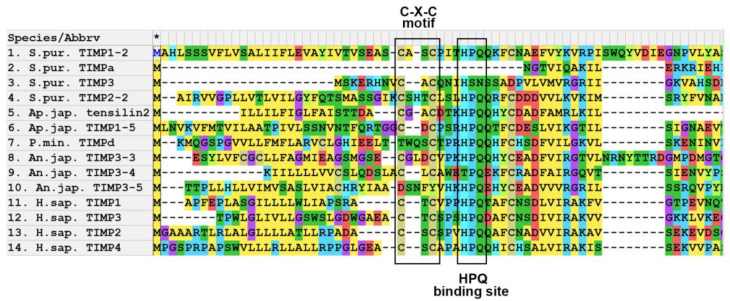
MUSCLE (MegaX) alignment of N-terminus of amino acid sequences of some TIMPs of echinoderms and human.

An important feature of TIMPs is the presence of the N-terminus C-X-C motif, in which one amino acid residue is located between the first and second cysteines (Figure 7). In vertebrates, such an amino acid residue is threonine (T) or serine (S). The function of this motive is to interact with a special section of the MMP, S1 pocket [84,85,130]. In mammals, the inclusion of an additional amino acid between the first and second cysteines leads to a disruption in the ability of TIMPs to bind to MMPs [76].

Most of the examined TIMPs of echinoderms also have an N-terminus C-X-C motif (Figure 7, Supplementary File 2). The amino acid residue located between the cysteines varies in different TIMPs even within the same species. This distinguishes TIMPs in echinoderms from TIMPs in vertebrates. In addition, echinoderms have several TIMP-like proteins, in which not one, but two, or even three amino acid residues are located between the first and second cysteines at the N-terminus (Figure 7) [35,78,79]. Some TIMP-like proteins lack the C-X-C motif and/or HPQ binding site (Figure 7).

### 3.2. Evolution of TIMP of Echinoderms

**Figure 8:**
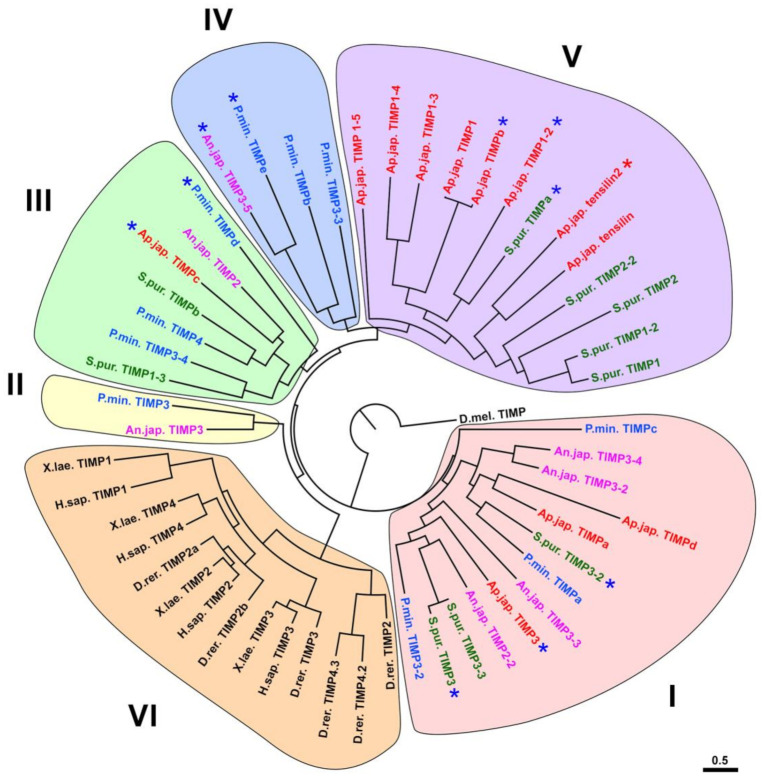
Phylogenetic tree showing the relationships of TIMPs of echinoderms. Crinoids (*Anneissia japonica*)—pink color, asteroids (*Patiria miniata*)—blue color, echinoids (*Strongylocentrotus purpuratus*)—green color, holothurians (*Apostichopus japonicus*)—red color, red asterisk—tensilin, blue asterisks—TIMP-like proteins that lack cysteines at the N-terminus of the molecule and/or the HPQ binding site. Construction of the phylogenetic tree was done using the MrBayes/Maximum Likelihood algorithm and the online service CIPRES (http://www.phylo.org, accessed on 15 March 2021). The nucleotide and amino acid sequences were analyzed using the Partitionfinder 2.1.1 [101,102]. The trees were visualized in the FigTree program.

Differences in the structure of the studied TIMPs of echinoderms are reflected in their location on the phylogenetic tree. All of them are clustered separately from the TIMPs of vertebrates (Figure 8). This confirms the previously obtained data on the evolution of these proteins [35,78]. The TIMPs of echinoderms can be divided into five groups. Group I is located closest to the base of the tree. It probably unites the most ancient TIMPs characteristic of the ancestral forms of deuterostomes. Orthologs of these genes were preserved in representatives of all classes of echinoderms, but apparently disappeared in vertebrates [78].

TIMPs included in group II are located closest to the TIMPs of vertebrates on the phylogenetic tree (group VI) (Figure 8). Apparently, they are all descendants of the gene that gave rise to all four mammalian TIMPs. Judging by the data of Clouse et al. [78], its orthologs are found not only in crinoids and asteroids (Figure 8), but also in other classes of echinoderms.

Groups III, IV, and V are unrelated to TIMPs in vertebrates and are probably descended from orthologs of genes formed after the separation of Chordata and Ambulacraria. TIMPs located in group III probably arose on the basis of one common ancestral gene, the descendants of which were preserved in all classes of echinoderms (Figure 8). Groups IV and V combine TIMPs of a later origin. Their divergence apparently took place within the phylum Echinodermata. Group IV contains only TIMPs of crinoids and asteroids, while group V assemble only echinoids and holothurians. At the same time, group V accumulates 2/3 of all holothurian TIMPs. This indicates that it is the TIMPs of this group that underwent the strongest duplication and divergence in the class Holothuroidea. This group also includes specific TIMP-like proteins—tensilins (Figure 8). These proteins are distinguished by the presence of two amino acid residues between the first and second cysteines of the N-terminal region of the molecule [35].

For the first time, tensilin was found in the connective tissue of the body wall of the holothurian *Cucumaria frondosa* (Gunnerus, 1767) [131]. It was assumed that such proteins are present in all echinoderms and are an important component of the mechanism of regulation of MCT properties [82,132]. However, phylogenetic analysis showed that all identified tensilin-like proteins belong to members of relatively young groups of holothurians [35,79]. No similar proteins are found in Apodida and Elasipodida (the most ancient orders of holothurians [133]), or in other echinoderms. Accordingly, the tensilins formed within the order Holothuroidea [35,78]. The ancestral gene of tensilin, apparently, was repeatedly duplicated, since some species of holothurians have several of its orthologs. For example, in *Cladolabes schmeltzii* two *tensilin* genes were identified [35], and in *E. fraudatix*—four [79]. Repeated duplication and preservation of orthologs in phylogenesis shows that tensilins play an important role in the physiology of holothurians.

As already mentioned, the studied species of echinoderms have TIMP-like proteins that lack cysteines at the N-terminus of the molecule and the HPQ binding site [78] (Figure 7, Supplementary File 2). However, the NTR domain is detected in them and the BLAST program identifies them as TIMPs. On the phylogenetic tree, these TIMP-like proteins are located in different groups (Figure 8). It is obvious that such proteins have arisen in the evolution of echinoderms repeatedly and independently of each other.

The reasons for the active duplication and divergence of *TIMPs* in the phylogenesis of Echinodermata are possibly associated with an increase in the functional importance of these proteins. Echinoderms are characterized by a high content of connective tissue in organs, as well as the presence of asexual reproduction and autotomy. In this regard, they need effective ECM remodeling mechanisms both for carrying out normal life activities (movement, changing posture and body shape), and for successful fission and separation of body parts during autotomy. The improvement of these mechanisms may have led to the emergence and preservation of the diversity of not only MMPs, but also TIMPs. In addition, an increase in the number of *TIMP* genes could also occur in connection with the functional divergence of the proteins encoded by them. It is known that in mammals, TIMPs can perform functions unrelated to MMP inhibition [134]. One of the directions of such divergence could be the formation of TIMP-like proteins in echinoderms, in particular, tensilins.

### 3.3. Functions

The functions of TIMPs in echinoderms have not been studied at all. It is obvious that, as in mammals, many of these proteins are MMP inhibitors. As mentioned above, MMPs play an important role in the mechanisms of development and regeneration in echinoderms. In this regard, TIMPs can interact with MMPs and, accordingly, regulate the activity of various biological processes, such as cell division, cell migration, ECM remodeling, etc. In particular, TIMP was discovered in the coelomic fluid of the asteroid *Asterias rubens* Linnaeus, 1758 [135]. The content of this protein increased six hours after the arm tip was cut off and the coelomic fluid was completely removed. It is possible that the increase in the quantity of TIMP was associated with the need to block protease activity for more successful thrombus formation on the wound surface.

As an MMP inhibitors, TIMPs in echinoderms may be involved in the regulation of MCT properties [78,82,132]. The TIMP-like protein, tensilin, is considered to be one of the key molecules of this process [132]. It is assumed that its function is to inhibit MMPs and prevent them from breaking down the crosslink complexes between collagen fibrils, which, in turn, increases the stiffness of connective tissue [82,132]. However, as shown earlier, tensilins are found only in species of the order Holothuroidea [35]. Probably, in other echinoderms, some TIMPs, possibly belonging to groups IV and V (Figure 8), are involved in the mechanisms of MCT regulation. In holothurians, due to the formation of a thick connective tissue body wall and a decrease in the degree of its calcification, MMPs involved in the destruction of crosslink complexes could be replaced by more efficient proteinases. This, in turn, could lead to a change in TIMPs inhibiting them and the formation of a specialized group of TIMP-like proteins—tensilins.

Another possible function of echinoderm TIMPs is participation in the regeneration. It has been shown that in the holothurian *E. fraudatrix* one of the *tensilin* genes, *Ef-tensilin3*, is expressed during gut regeneration [79]. In the early stages of regeneration its transcripts were found in the coelomic epithelium of the mesentery and gut anlage. The most intense expression was located in the ventral part of the forming digestive tube. In the course of regeneration, the intensity of the expression decreased. On 10^th^ day after damage, the highest expression of this gene occurred only in the growing tip of the gut and in the ventral part of the luminal epithelium. In holothurians, during the development of the anlage of the digestive tube, collagen is synthesized and accumulated in the ventral edge of the intestinal mesentery [17,18,19,32,33,55,81,136,137]. It is likely that blocking MMP activity by *Ef-tensilin3* is necessary to stabilize the extracellular matrix and form the base for the digestive tube.

It should be noted that mammalian TIMPs, in addition to inhibiting MMPs, are involved in a wide range of biological functions [134,138,139,140]. For example, TIMP-1, interacting with MT1-MMP, helps to activate pro-MMP2 [141]. This mechanism stimulates cell migration and enhances tumor metastasis. TIMPs show cell growth promoting activity and can modulate cell apoptosis regardless of their MMP inhibitory activity [142,143,144,145]. TIMP-1 and TIMP-2 have been described to inhibit the proliferation of cells including endothelial cells and several carcinoma cells [142,146]. In addition, mammalian TIMP-1 is able to bind to CD63 and integrins and regulate cell survival and polarization, as well as modulate FAK/RhoA signaling [147,148]. In this regard, it can be assumed that the TIMPs of echinoderms, in addition to inhibiting MMPs, can also perform other functions.

## 4. Conclusions

Analysis of the available genomes and existing literature showed that echinoderms have a wide variety of *MMP* and *TIMP* genes. This indicates the prominent role of the proteins encoded by them in the physiology of these animals. The *MMP* and *TIMP* genes underwent repeated duplication and active divergence after the separation of Ambulacraria and Chordata, as a result of which the homology of proteinases and their inhibitors between these groups cannot be established. Special studies are needed to develop a classification of MMPs and TIMPs in echinoderms.

Nevertheless, the MMPs and TIMPs of echinoderms and vertebrates have retained much in common. They have a similar domain structure and, apparently, a similar function. Echinoderm proteinases can be structurally divided into three groups—archetypal MMPs, matrilysins, and furin-activatable MMPs. Echinoderms, apparently, do not have gelatinases homologous to those of vertebrates. The main function of MMPs in echinoderms is the degradation of various proteins. In this regard, they play an important role in the mechanisms of development, asexual reproduction and regeneration.

TIMPs of echinoderms, with a few exceptions, have a structure typical for this class of proteins. They contain an NTR domain and 10–12 conservatively located cysteine residues, which probably form the tertiary structure of the molecule. At the same time, the structure of TIMPs in echinoderms shows greater diversity than in vertebrates. In the phylogenesis of echinoderms, the ancestral TIMP genes underwent significant divergence and repeated duplication, which was probably associated with an increase in the functional importance of the proteins encoded by them.

## Supplementary Materials

The following are available online at https://www.mdpi.com/article/10.3390/cells10092331/s1, Supplementary Files 1 and 2: The sequences used for analysis; Supplementary File 3: A list of protein names and accession numbers of nucleotide sequences of the corresponding transcripts from the NCBI.
