# Supplementary material for: Matrix Metalloproteinases and Tissue Inhibitors of Metalloproteinases in Echinoderms: Structure and Possible Functions
Source: Cells. 2021 Sep 6;10(9):2331. doi: 10.3390/cells10092331 (PMC8467561; doi:10.3390/cells10092331)
Supplement: Supplementary file 1 [file cells-10-02331-s001.zip › Suppl3_(Seq_list).pdf]

***Anneissia japonica* (PRJNA615663)**
An.jap. _hatching enzyme_ - XM_033258775.1
An.jap. _MMP1-1_ - XM_033246259.1
An.jap. _MMP2_ - XM_033270892.1
An.jap. _MMP2-2_ - XM_033270859.1
An.jap. _MMP2-3_ - XM_033252596.1
An.jap. _MMP2-4_ - XM_033270828.1
An.jap. _MMP3_ - XM_033268939.1
An.jap. _MMP7_ - XM_033239646.1
An.jap. _MMP8_ - XM_033267661.1
An.jap. _MMP11_ - XM_033270068.1
An.jap. _MMP13_ - XM_033239630.1
An.jap. _MMP14_ - XM_033271712.1
An.jap. _MMP15_ - XM_033252512.1
An.jap. _MMP15-2_ - XM_033270829.1
An.jap. _MMP16-1_ - XM_033252624.1
An.jap. _MMP16-2_ - XM_033270854.1
An.jap. _MMP17_ - XM_033246263.1
An.jap. _MMP18_ - XM_033252622.1
An.jap. _MMP18-2_ - XM_033252621.1
An.jap. _MMP18-3_ - XM_033239647.1
An.jap. _MMP21_ - XM_033250918.1
An.jap. _MMP24_ - XM_033252607.1

An.jap_TIMP2_ - XP_033106593.1
An.jap_TIMP2-2_ - XP_033106529.1
An.jap_TIMP3_ - XP_033106552.1
An.jap_TIMP3-2_ - XP_033106571.1
An.jap_TIMP3-3_ - XP_033106568.1
An.jap_TIMP3-4_ - XP_033106476.1
An.jap_TIMP3-5_ - XP_033126188.1

***Patiria miniata* (PRJNA683060)**
P.min. _hatching enzyme_ - XM_038190972.1
P.min. _hatching enzyme-2_ - XM_038204489.1
P.min. _MMP2_ - XM_038203824.1
P.min. _MMP2-2_ - XM_038198954.1
P.min. _MMP7_ - XM_038200528.1
P.min. _MMP9_ - XM_038209867.1
P.min. _MMP13_ - XM_038219104.1
P.min. _MMP14_ - XM_038210303.1
P.min. _MMP14-1_ - XM_038200576.1
P.min. _MMP14-2_ - XM_038200551.1
P.min. _MMP14-3_ - XM_038191009.1
P.min. _MMP16_ - XM_038200539.1
P.min. _MMP16-2_ - XM_038210989.1
P.min. _MMP16-3_ - XM_038204118.1

P.min. _MMP17_ - XM_038200564.1
P.min. _MMP18_ - XM_038209880.1
P.min. _MMP18-2_ - XM_038199580.1
P.min. _MMP19_ - XM_038204488.1
P.min. _MMP24_ - XM_038210053.1
P.min. _MMP24-2_ - XM_038209763.1

P.min_TIMPa_ - XP_038060798.1
P.min_TIMPb_ - XP_038060798.1
P.min_TIMPc_ - XP_038060882.1
P.min_TIMPd_ - XP_038060881.1
P.min_TIMPe_ - XP_038049368.1
P.min_TIMP3_ - XP_038060714.1
P.min_TIMP3-2_ - XP_038060714.1
P.min_TIMP3-3_ - XP_038060669.1
P.min_TIMP3-4_ - XP_038062057.1
P.min_TIMP4_ - XP_038061469.1

***Strongylocentrotus purpuratus* (PRJNA13728)**
S.pur. _hatching enzyme_ - XM_001176086.4
S.pur. _MMP1_ - XM_003726184.3
S.pur. _MMP1-2_ - XM_030985841.1
S.pur. _MMP2_ - XM_030984783.1
S.pur._MMP2-2_ - XM_030980005.1
S.pur. _MMP2-3_ - XM_785589.5
S.pur. _MMP2-4_ - XM_030980843.1
S.pur. _MMP7_ - XM_030980004.1
S.pur. _MMP9_ - XM_003728326.2
S.pur. _MMP11_ - XM_031000493.1
S.pur. _MMP11-2_ - XM_030972234.1
S.pur. _MMP12_ - XM_786507.5
S.pur. _MMP12-2_ - XM_786523.5
S.pur. _MMP13_ - XM_030984127.1
S.pur. _MMP14_ - XM_030980002.1
S.pur. _MMP16_ - NM_001033648.1
S.pur. _MMP16-2_ - XM_781452.5
S.pur. _MMP17_ - XM_030979866.1
S.pur. _MMP18_ - XM_783693.4
S.pur. _MMP21_ - XM_003729663.3
S.pur. _MMP24_ - XM_003725508.3
S.pur. _MMP24-2_ - XM_030992226.1

S.pur_TIMPa_ - XP_030828995.1
S.pur_TIMPb_ - XP_003728985.1
S.pur_TIMP1_ - XP_003725524.1
S.pur_TIMP1-2_ - XP_001198302.2
S.pur_TIMP1-3_ - XP_030829173.1

S.pur_TIMP2_ - XP_003725525.2
S.pur_TIMP2-2_ - XP_030828942.1
S.pur_TIMP3_ - XP_011673577.1
S.pur_TIMP3-2_ - XP_030828686.1
S.pur_TIMP3-3_ - XP_003725523.1

Apostichopus japonicus **(PRJNA354676)**
Ap.jap. _MMP1_ - KR872413.1
Ap.jap. _MMP2-1_ - MH348178.1
Ap.jap. _MMP2-2_ - MRZV01000410.1
Ap.jap. _MMP2-3_ - MRZV01000538.1
Ap.jap. _MMP2-4_ - KX372219.1
Ap.jap. _MMP12_ - MRZV01000538.1
Ap.jap. _MMP14_ - PIK52473.1
Ap.jap. _MMP14-2_ - MRZV01000664.1
Ap.jap. _MMP14-3_ - PIK37483.1
Ap.jap. _MMP16_ - MG009443.1
Ap.jap. _MMP16-2_ - MRZV01000419.1
Ap.jap. _MMP16-3_ - KX372220.1
Ap.jap. _MMP16-4_ - MRZV01001258.1
Ap.jap. _MMP19_ - MRZV01000751.1
Ap.jap. _MMP21_ - MRZV01001523.1
Ap.jap. _MMP24_ - MRZV01001258.1
Ap.jap. _hypothetical protein BSL780-00943_ - MRZV01000018.1
Ap.jap. _hypothetical protein BSL78-16904_ - MRZV01000658.1

Ap.jap_TIMPa_ - PIK53955.1
Ap.jap_TIMPb_ - ALL53534.1
Ap.jap_TIMPc_ - PIK35025.1
Ap.jap_TIMPd_ - PIK51337.1
Ap.jap_TIMP1_ - PIK53592.1
Ap.jap_TIMP1-2_ - PIK53588.1
Ap.jap_TIMP1-3_ - PIK53589.1
Ap.jap_TIMP1-4_ - AVQ67918.1
Ap.jap_TIMP1-5_ - PIK35024.1
Ap.jap_TIMP3_ - PIK53586.1
Ap.jap_tensilin_ - PIK53591.1
Ap.jap_tensilin2_ - PIK52999.1

Saccoglossus kowalevskii **(PRJNA42857)**
S.kow. _MMP14_ - XM_002740999.2
S.kow. _MMP14-2_ - XM_006824319.1
S.kow. _MMP14-3_ - XM_006825494.1
S.kow. _MMP19_ - XM_002730707.2
S.kow. _MMP19-2_ - XM_002738541.2
S.kow. _MMP19-3_ - NM_001184851.1
S.kow. _MMP19-4_ - XM_006812184.1

S.kow. _MMP19-5_ - XM_002731281.2
S.kow. _MMP21_ - XM_006815506.1
S.kow. _MMP24_ - XM_002738540.2

***Danio rerio* (PRJNA13922)**
D.rer. _MMP2_ - NM_198067.1
D.rer. _MMP9_ - NM_213123.1
D.rer. _MMP11a_ - XM_021478463.1
D.rer. _MMP11b_ - XM_689061.9
D.rer. _MMP13a_ - XM_009305556.3
D.rer. _MMP13b_ - XM_001345471.7
D.rer. _MMP14a_ - NM_194416.1
D.rer. _MMP14b_ - XM_005171310.4
D.rer. _MMP15a_ - XM_001924007.7
D.rer. _MMP15b_ - XM_021467433.1
D.rer. _MMP16b_ - XM_695689.9
D.rer. _MMP17a_ - XM_693509.9
D.rer. _MMP17b_ - NM_001365502.1
D.rer. _MMP18_ - XM_021479446.1
D.rer. _MMP19_ - XM_021479982.1
D.rer. _MMP20a_ - XM_017351189.2
D.rer. _MMP20b_ - XM_009291754.3
D.rer. _MMP21_ - NM_001317753.1
D.rer. _MMP23ba_ - XM_021478518.1
D.rer. _MMP23bb_ - NM_001017890.1
D.rer. _MMP24_ - XM_694711.7
D.rer. _MMP25a_ - XM_021471453.1
D.rer. _MMP25b_ - XM_002663896.5
D.rer. _MMP28_ - XM_001337545.7
D.rer. _MMP30_ - NM_001040291.1

D.rer_TIMP2_ - XP_005172816.1
D.rer_TIMP2a_ - NP_878294.1
D.rer_TIMP2b_ - NP_998461.1
D.rer_TIMP3_ - XP_005174719.3
D.rer_TIMP4.2_ - NP_001315189.1
D.rer_TIMP4.3_ - NP_001313362.1

***Xenopus laevis* (PRJNA338693)**
X.lae. _MMP1S_ - NM_001094954.1
X.lae. _MMP2S_ - NM_001087228.2
X.lae. _MMP3_ - XM_018247760.1
X.lae. _MMP3-1_ - XM_018250308.1
X.lae. _MMP3L_ - NM_001093594.1
X.lae. _MMP7_ - NM_001086393.1
X.lae. _MMP7L_ - NM_001086213.1
X.lae. _MMP8L_ - NM_001087049.2

X.lae. _MMP8S_ - NM_001095034.2
X.lae. _MMP9_ - XM_018238374.1
X.lae. _MMP9-2L_ - NM_001097836.1
X.lae. _MMP9S_ - NM_001086503.1
X.lae. _MMP11L_ - XM_018262938.1
X.lae. _MMP13S_ - NM_001086405.1
X.lae. _MMP14_ - XM_018243932.1
X.lae. _MMP15_ - XM_018260625.1
X.lae. _MMP16_ - XM_018223848.1
X.lae. _MMP17_ - XM_018244170.1
X.lae. _MMP17-2_ - XM_018236930.1
X.lae. _MMP18_ - XM_018247758.1
X.lae. _MMP18-2_ - XM_018245742.1
X.lae. _MMP18-3_ - XM_018247759.1
X.lae. _MMP18-4_ - XM_018248750.1
X.lae. _MMP18-5_ - XM_018245743.1
X.lae. _MMP18-6_ - XM_018245744.1
X.lae. _MMP18-7_ - XM_018248751.1
X.lae. _MMP18-8_ - XM_018231261.1
X.lae. _MMP20L_ - NM_001097975.1
X.lae. _MMP21_ - XM_018254889.1
X.lae. _MMP21-2_ - XM_018241577.1
X.lae. _MMP21L_ - NM_001085816.1
X.lae. _MMP28L_ - NM_001172229.2
X.lae. _MMP28S_ - NM_001090135.1

X.lae_TIMP1_ - XP_018087965.1
X.lae_TIMP2_ - XP_018093841.1
X.lae_TIMP3_ - NP_001079064.1
X.lae_TIMP4_ - XP_018095919.1

***Homo sapiens* (PRJNA168)**
H.sap. _MMP1_ - BT020147.1
H.sap. _MMP2_ - NM_001127891.3
H.sap. _MMP3_ - NM_002422.4
H.sap. _MMP7_ - NM_002423.4
H.sap. _MMP8_ - NM_001304441.1
H.sap. _MMP9_ - NM_004994.2
H.sap. _MMP10_ - NM_002425.2
H.sap. _MMP12_ - NM_002426.5
H.sap. _MMP13_ - NM_002427.3
H.sap. _MMP14_ - NM_004995.3
H.sap. _MMP15_ - NM_002428.3
H.sap. _MMP16_ - NM_005941.4
H.sap. _MMP17_ - NM_016155.6
H.sap. _MMP18_ - Y08622.1
H.sap. _MMP20_ - NM_004771.3

H.sap. _MMP21_ - AF520613.1
H.sap. _MMP23_ - AJ005256.1
H.sap. _MMP24_ - NM_006690.3
H.sap. _MMP25_ - NM_022468.4
H.sap. _MMP26_ - NM_021801.4
H.sap. _MMP27_ - NM_022122.2
H.sap. _MMP28_ - BC002631.2

H.sap_TIMP1_ - NP_003245.1
H.sap_TIMP2_ - NP_003246.1
H.sap_TIMP3_ - NP_000353.1
H.sap_TIMP4_ - NP_003247.1

***Drosophila melanogaster* (PRJNA164)**
D.mel. _MMP1_ - NM_001259570.2
D.mel_TIMP_ - NP_731461.1
